# Quasi-Experimental designs for quality improvement research

**DOI:** 10.1186/1748-5908-8-S1-S3

**Published:** 2013-04-19

**Authors:** Alene Toulany, Rory McQuillan, Jennifer D Thull-Freedman, Peter A Margolis

**Affiliations:** 1 Department of Paediatrics, Division of Adolescent Medicine, The Hospital for Sick Children Toronto, Ontario, M5G 1X8, Canada; 2 Division of Nephrology, University Health Network, Toronto General Hospital, Ontario, M5G 2C4, Canada; 3 Department of Pediatrics, University of Calgary; Alberta Children's Hospital, Calgary, Alberta, T3B 6A8, Canada; 4 James M. Anderson Center for Health System Excellence, Division of Health Policy and Clinical Effectiveness, Cincinnati Children's Hospital Medical Center, Cincinnati, Ohio, 45229, USA

## Presentation

Quality Improvement (QI) research may be defined as “the design, development and evaluation of complex interventions aimed at the re-design of health care systems to produce improved outcomes”. The challenge of QI lies in bridging the gap between knowing what needs to happen at an individual patient level and implementing this at a systems level. The inherent complexity of systems poses challenges in terms of implementation, but also presents the researcher with circumstances for which conventional research methods may not prove useful.

Explanatory trials are designed to answer the question “does this intervention work under ideal circumstances?” Patient and system variability are typically rigorously controlled. Pragmatic trials seek to answer how well an intervention works in usual practice [1]. It is important to contend with variation (e.g., in patient volume or complexity) and not control for it. Consider the analogy of water sampled from a pond versus a river [2]. If one takes random samples of water from a still pool of water one can draw inference about the pond as a whole, as it is relatively static and unchanging. This is the principle we are using in attempting to extrapolate the findings of a randomized controlled trial to a population. The real world however, behaves far more like a river where the water changes from second to second, influenced by innumerable complex interacting factors such as the season, rain, construction.

In QI research it is important to understand the changing nature of the river (i.e. causes of system variation) in order to be able to predict how to make an intervention work under all the conditions in which it will be expected to perform.

QI research should focus therefore on robust, sequential experimentation. Too often, quality improvement investigators seek to proceed to clinical trials before sufficient exploration, investigation, and understanding of the complex system and its interactions have been achieved.

Campbell et al present a trajectory for QI research required to build requisite knowledge [3]. The design and testing of complex interventions in care delivery proceeds through a series of planned stages. One begins by developing a concept or theory and then progresses to designing a prototype. Next, an intervention is piloted on a small scale before performing a detailed test and finally disseminating the ideas generated. A variety of study designs may be used as learning proceeds across this trajectory of understanding. Research methods that address issues of internal validity without randomization of individuals are referred to as “quasi-experimental” designs and include time-series, equivalent time series, multiple baseline and factorial design.

## Commentary

In order to successfully implement change or study improvement initiatives, the researcher should understand the unique nuances of the system in which a change will operate. QI investigators should seek to understand why a change works or doesn’t work in a given context, rather than simply determining the effect while treating contextual factors as features to be held constant. It is helpful for studies to include measures contextual factors (e.g., team skills, microsystem motivation, leadership, and organizational culture and strategic priorities in improving health care systems). Without more defined and detailed descriptions of local contexts, we cannot build on knowledge learned across systems improvement initiatives. Important lessons can be learned from both high and low performing teams. Furthermore, we must not forget the importance of looking outside of our own disciplines for insights. Fields of engineering, business, psychology, and social sciences offer methodological approaches that are particularly relevant to QI research.

## Recommendations

Several barriers exist to large-scale QI research, including needing many participating sites when the unit of analysis is the site; having a limited number of outcomes at a given site, and the challenge of managing improvement interventions across sites. Research infrastructure such as formal training of investigators in the design of complex, multi-factor experiments and “labs” capable of supporting experimentation across units of analysis (patient, practice, health care system) will address some of these challenges. We recommend building research programs capable of supporting experimentation at all units of analysis to help advance the field of quality improvement research [4]. Sequential experimentation needs to occur at the patient, practice, and health system levels.

How might this be done in the “real world”? Start by thinking of a clinic or ward as such a clinical laboratory. An initial step is to standardize processes of interest within the microsystem. Choose methodologies that allow measurement and understanding of context. Focus on problems relevant to the frontlines. Leaders and organizations may be willing to provide funding through the operating budgets, for innovations in care delivery that are aligned with the strategic plan of the institution and may be cost-effective. In addition to building laboratories, attention should be focused on building and strengthening networks of clinical sites that can work cooperatively on QI studies.

A change in the medical scientific culture will be required to shift from trials designed to reduce complexity, towards more robust experimentation that incorporates system complexity directly. Much work remains to be done to develop methods that will enable effective and efficient learning in complex systems where quality improvement takes place.
